# Evolutionary Dynamics of Fearfulness and Boldness: A Stochastic Simulation Model

**DOI:** 10.1371/journal.pone.0032258

**Published:** 2012-03-08

**Authors:** Nan Lu, Ting Ji, Jia-Hua Zhang, Yue-Hua Sun, Yi Tao

**Affiliations:** 1 Group of Theoretical Biology, Group of Avian Ecology, Key Laboratory of Animal Ecology and Conservation Biology, Institute of Zoology, Chinese Academy of Sciences, Beijing, P.R. China; 2 Graduate University of Chinese Academy of Sciences, Beijing, P.R. China; University of Sheffield, United Kingdom

## Abstract

A stochastic simulation model is investigated for the evolution of anti-predator behavior in birds. The main goal is to reveal the effects of population size, predation threats, and energy lost per escape on the evolutionary dynamics of fearfulness and boldness. Two pure strategies, fearfulness and boldness, are assumed to have different responses for the predator attacks and nonlethal disturbance. On the other hand, the co-existence mechanism of fearfulness and boldness is also considered. For the effects of total population size, predation threats, and energy lost per escape, our main results show that: (i) the fearful (bold) individuals will be favored in a small (large) population, i.e. in a small (large) population, the fearfulness (boldness) can be considered to be an ESS; (ii) in a population with moderate size, fearfulness would be favored under moderate predator attacks; and (iii) although the total population size is the most important factor for the evolutionary dynamics of both fearful and bold individuals, the small energy lost per escape enables the fearful individuals to have the ability to win the advantage even in a relatively large population. Finally, we show also that the co-existence of fearful and bold individuals is possible when the competitive interactions between individuals are introduced.

## Introduction

Individuals within a single local population of the same vertebrate species differ in their propensity to take risks [1], [2], and these differences in a range of correlated behavioral traits have also been labeled as animal personality [2], [3], or behavioral syndromes [4], [5]. Furthermore, animals often show very limited behavioral plasticity and commonly differ consistently in their reaction towards the same environmental stimulus [1], [2], [4]–[6] These differences have already been indicated to have a substantial genetic basis that can be inherited from generation to generation [7]–[10]. For birds, fearfulness-boldness as an anti-predator behavior continuum varies among different species or populations [11], [12], and should partly ascribe to the dissimilar evolutionary history [13], [14].

Recently, two theoretical evolutionary game models are developed to explain how birds respond to the predation threat, i.e. the evolution of fearfulness and boldness [15], [16]. When a bird flock is threatened, birds can not immediately identify whether it is a real attack or not, the fearful bird will take flight immediately anyhow, but the bold one will on alert for some time and take flight only if the threat proves to be a real attack [15]. Therefore, there will be a trade-off between survival and reproduction [15], [16]. The fearful individuals have more chances to survive, but will have less energy left for reproduction due to more energy consumptions through taking flight than the bold one. Sirot demonstrated that the predicted levels of fearfulness are extremely variable depending on the respective frequencies of predatory attacks and simple disturbing events, and on the capacity of birds to detect and escape predators [15]. However, Ji et al. found that the simple coexistence of two pure strategies (i.e. fearfulness and boldness) is surprisingly impossible, and a small population is favorable to fearful individuals, while boldness is preferred in a large population [16]. Furthermore, Ji et al. showed also that the existence of a mixed ESS strategy is impossible [16]. They explained that such phenomenon may ascribe to the ‘dilution effects’, i.e. individuals are safer because each individual in a large population has a relatively smaller chance of being the one attacked [17]. Specifically, bold individuals will have a higher expected fitness in a large population than in a small one due to the declined predation risk and less flying energy loss [16]. Nonetheless, it is still not clear that how the dilution or risk sharing effects act on the evolutionary process, and the sensitivity of such effects also remains to be explored. Therefore, in this paper, we develop a stochastic simulation model with overlapping generations to investigate the evolution of fearfulness and boldness, and our main goal is to illustrate the effects of population size, predation risks and energy lost per escape on evolutionary dynamics of fearfulness and boldness. On the other hand, since the maintenance of variation in personality in natural populations are still largely unknown [18], a possible mechanism for the co-existence of fearfulness and boldness will be also developed through introducing the interactions between individuals.

## Methods

### Assumptions and model

Following Sirot and Ji et al. [15], [16], in order to explore the evolutionary dynamics of fearfulness and boldness in a bird population, a stochastic simulation model is developed. Here, for simplicity, we consider an asexual population undergoing both predatory attacks and non-lethal disturbing events [16]. Only two possible behavior traits can be exhibited when the population is disturbed, one is fearfulness (denoted by 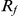) and the other boldness (denoted by 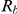). According to Sirot [15], the two phenotypes 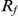 and 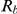 are defined as “when the population is disturbed, fearful individuals take escape immediately, but bold individuals are on the alert for some time and then take escape only if the threat proves to be a real predator attack.” This definition also implies that when the population is under predator attacks, a fearful individual should have more chances for survival since it always leaves early, but this may be unfavorable for its reproductive success because of the energy lost [15], [16], [19] . For our model, the other definitions and assumptions are given below:

- All individuals in the population are pure strategists. The number of 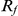-individuals is denoted by 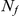, and the number of 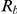-individuals by 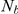. The total population size is denoted by 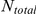, i.e. 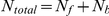, and we further assume that 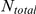 is kept to be a constant at the end of each breeding season.
- The generations are overlapping. For both phenotypes 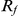 and 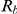, all individuals are assumed to have the same maximum natural life (or maximum survival age), denoted by 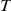 year old. The individual's maturity age for reproduction is one year old, and the offspring will have the same phenotype with their mother [16], [20].
- During a breeding season, the number of real predatory attacks is assumed to be a constant, denoted by 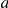, and, similarly, the number of simple disturbing events is denoted by 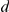. In order to show the change in the number of individuals during a breeding season, let 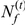 and 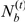 denote the numbers of 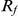
- and 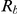-individuals at the starting of 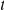-th breeding season, respectively. From Sirot [15] and Ji et al. [16], let 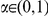 denote the relative probability that a 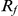-individual is selected by the predators, compared with a 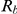-individual. This means that if 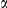 is near 0 then the 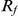-individuals are almost never attacked; conversely, if 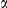 is near 1, then the risk is shared more equally by both 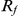
- and 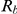
- individuals. We also use 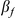 to denote the probability that a 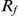-individual is captured when selected by the predator, and 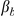 the probability that a 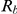-individual is captured when selected. Thus, the probabilities that the fearful individuals are selected by the predators and a single 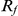-individual is killed at the 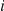-th attack are given by

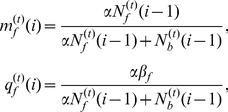
(1)where 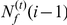 and 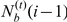 are the numbers of 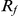
- and 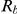-individuals after the 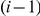-th attack, respectively. Similarly, the probabilities that the bold individuals are selected by the predators and a single 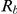-individual is killed at the 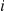-th attack are given by
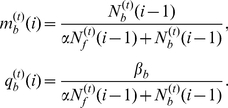
(2)

- Assume that the reproduction only occurs at the end of each breeding season. During a breeding season, if a 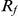-individual survives to the time of reproduction, then the level of its energy reserves can be simply expressed as 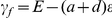, where 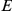 represents the total energy gained during a breeding season, and 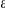 is the energy lost per escape. Similarly, if a 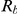-individual survives to the time of reproduction, then the level of its energy reserves is 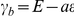. However, the reproductive success of an individual is proportional to the level of its energy reserves in general. From Sirot [15] and Ji et al. [16], the reproductive success of an individual with energy reserves 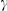 can be measured by the function 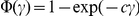 where 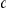 is a constant. This means that the reproductive success of a 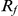-individual is

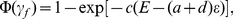
(3)and the reproductive success of a 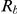-individual is
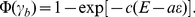
(4)

- Let 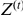 denote the total number of the dead individuals at the time of reproduction in the 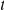-th breeding season due to the predator attacks and the limitation of individual's lifespan (i.e. the individuals with age 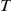 will be eliminated from the population at the end of the 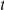-th breeding season even if these individuals are not killed by the predators). Since the total population size, 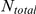, is assumed to be fixed at the end of each breeding session, the total number of offspring born in the 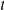-th breeding season should be exactly equal to 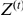. According to this definition, in the 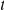-th breeding season, the expected number of 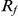-offspring, denoted by 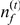, is

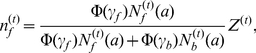
(5)where 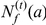 and 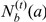 are the numbers of 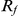
- and 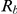-individuals after the 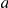-th attack, respectively (see also Eqs. 1 and 2), and similarly, the expected number of 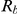-offspring, denoted by 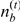, is
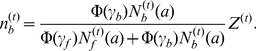
(6)

- However, in (i)-(v), the interactions between 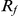
- and 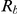-individuals, i.e. the background fitnesses of 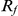
- and 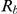-individuals, are ignored. Here, in order to show the effect of the background fitnesses on the evolutionary dynamics of 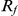 and 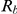, the background fitnesses of 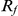
- and 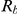-individuals, denoted by 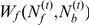 and 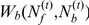, respectively, are defined by analogy to Lotka-Volterra interspecific competition as

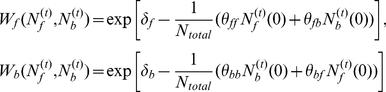
(7)(see Refs. [16], [21]), where 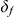 and 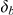 are constants, 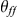 and 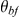 represent the effects of 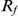-individuals on themselves and 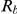-individual, respectively, and, similarly, 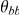 and 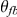 represent the effects of 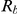-individual on themselves and 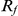-individuals, respectively. So, under this definition, the expected numbers of 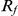
- and 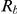-offspring in the 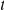-th breeding season (see Eqs. 5 and 6) can be rewritten as
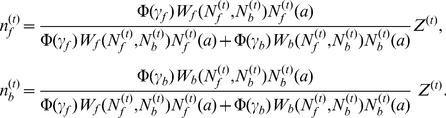
(8)


### Stochastic simulation

According to the definitions and assumptions in section 2, the stochastic simulation is conducted:

1. At the starting of 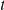-th breeding season, the number of 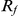-individuals with age 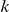 is denoted by 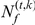 for 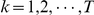, i.e. 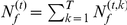, and, similarly, the number of 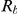-individuals with age 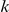 is denoted by 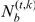 for 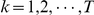, i.e. 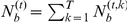.
2. The probability that a 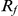-individual is killed by the predators at the 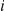-th attack is 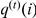 for 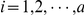 (see Eq. 1), and the probability that a 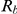-individual is killed by the predators at the 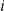-th attack is 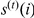 for 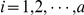 (see Eq. 2), where the numbers of 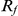
- and 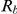-individuals with age 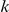 after 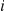-th attack are denoted by 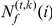 and 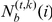, respectively, for 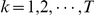 (i.e. 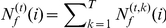 and 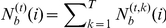) (see the assumption (iii) in section 2).
3. At the end of the 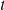-th breeding season, the total number of dead individuals at the time of reproduction is 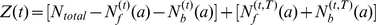, where the term 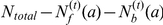 represents the number of dead individuals because of the predator attacks and the term 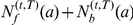 is the total number of individuals with age 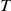 after the 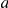-th attack (see the assumption (v) in section 2).
4. The numbers of new born 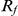
- and 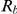-individuals at the end of the 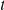-th breeding season are given by Eqs. 5 and 6, respectively (see also the assumption (v) in section 2).

For given the initial condition (i.e. the initial proportions of 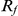
- and 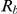-individuals), we run the simulation until the population becomes a pure strategy population (i.e. 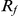-population, or 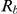-population). We repeat this process 1000 times and then count the times that the 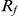-population occurs, denoted by 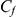, or the frequency that the 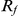-population occurs, denoted by 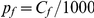.

Finally, in order to make our model (an individual-based model) to be understood well and to be tested, a standard ODD protocol [20] is given in Appendix S1, and the stochastic simulation program (i.e. simulation code) in Matlab is also provided in Appendix S1.

## Results

In this section, according to basic definitions and assumptions (i)–(v) (where the background fitnesses of fearful and bold individuals are ignored), we consider first the effects of the total population size, intensity of predator attacks and energy loss per escape on the evolutionary dynamics of fearfulness and boldness. Finally, according to assumption (vi), the effect of competitive interactions between fearful and bold individuals (i.e. background fitness) on the co-existence of fearfulness and boldness is considered.

### Effect of population size

**Figure 1:**
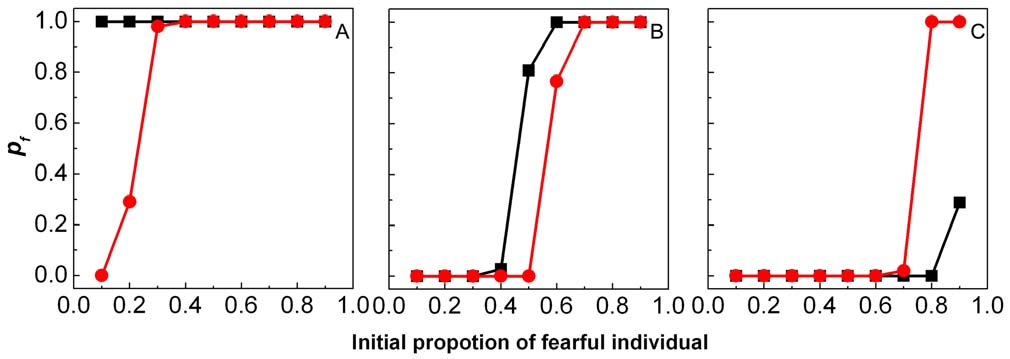
The effect of population size on the evolution of fearfulness and boldness. The population sizes are taken as 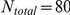 (A), 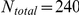 (B), and 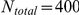 (C), respectively. 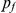 denotes the frequency that 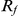-population occurs, i.e. 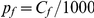. Simulations are conducted for both frequency-dependent (black line) and frequency-independent (red line) risk sharing situations.

We here set three levels for the total population size, which are 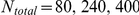, and the other parameters are taken as 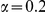, 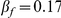, 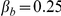, 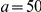, 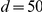, 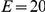 and 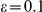. The simulation results are plotted in Figure 1, where the 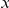-axis denotes the initial proportion of 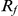-individuals, and the 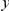-axis the frequency 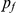. For the situation with that both 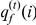 and 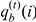 are frequency-dependent (see Eqs. 1 and 2), we have (i) for 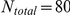, 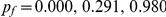 when the initial proportion of 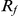 is 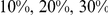, respectively, and 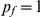 if the initial proportion of 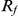 is equal to or bigger than 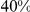 (Figure 1A, the red curve); (ii) for 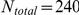, 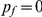 if the initial proportion of 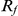 is equal to or less than 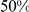, 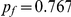 when the initial proportion of 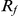 is 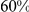, and 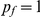 if the initial proportion of 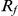 is equal to or bigger than 70% (Figure 1B, the red curve); and (iii) for 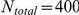, 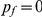 if the initial proportion of 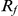 is equal to or less than 60%, 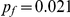 when the initial proportion of 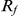 is 70%, and 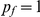 if the initial proportion of 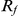 is equal to or bigger than 80% (Figure 1C, the red curve). These simulation results show clearly that for the situation with that both 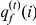 and 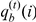 are frequency-dependent, the theoretical results [16] should be correct , i.e. the fearful individuals are favored in the small population, but the bold individuals will be advantageous in the large population.

On the other hand, we also consider the situation where we assume that both 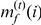 and 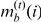 are independent of the numbers of 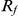
- and 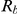-individuals, or that the probability that the fearful individuals (or bold individuals) are selected by the predators is independent of the population structure (i.e. it is frequency-independent). Then, for all possible 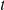 and 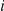, we have
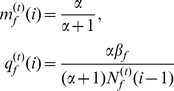
(9)and
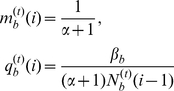
(10)(see Eqs. 1 and 2). Comparing with Eqs. 1 and 2, it is easy to see that
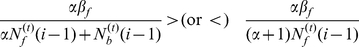
if 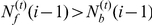 (or 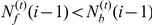), and, similarly,
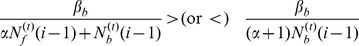
if 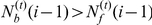 (or 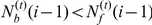). This implies that when the number of 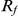-individuals is less than the number of 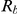-individuals, the risk of the 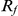-individuals will be shared by the 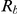-individuals, and, similarly, when the number of 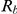-individuals is less than the number of 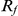-individuals, the risk of the 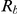-individuals will be shared by the 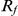-individuals.

For this situation,we have (i) for 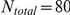, 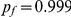 for initial proportions of 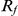 is equal to 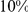, and 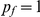 for all initial proportions of 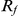 is equal to or bigger than 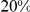 (Figure 1A, the black curve); (ii) for 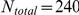, 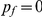 if the initial proportion of 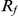 is equal to or less than 30%, 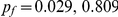 when the initial proportion of 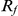 is 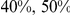, respectively, and 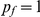 if the initial proportion of 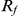 is equal to or bigger than 60% (Figure 1B, the black curve); and (iii) for 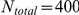, 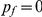 if the initial proportion of 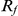 is equal to or less than 80%, and 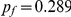 when the initial proportion of 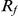 is 90% (Figure 1C, the black curve). It is easy to see that for 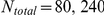, the fearful individuals are more advantageous in the situation with frequency-dependence than in the situation with frequency-independence, but, for 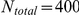, the fearful individuals more advantageous in the situation with frequency-independence than in the situation with frequency-dependence.

All of above results imply that when the population size is small, the risk that the bold individuals will be captured by the predators cannot be compensated by bold individuals' advantage in reproduction, i.e. when the population size is small, a single 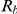-individual cannot invade successfully a 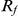-population; conversely, when the population size is large, the disadvantage of fearful individuals in reproduction cannot be compensated by their advantage in survival, i.e. when the population size is large, a single 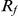-individual cannot invade successfully a 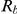-population.

### Effect of the intensity of predator attacks

**Figure 2:**
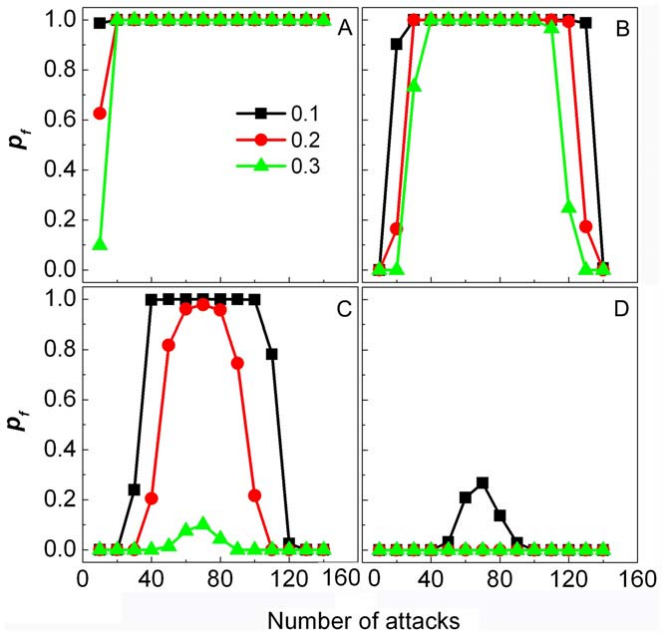
The effects of the intensity of real predatory attacks and population size on the evolution of fearfulness and boldness where 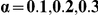. 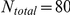 (A), 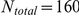 (B), 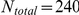 (C), and 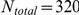 (D).

In this subsection, the effect of the intensity of predators attacks (i.e. the number of predators attacks) on the system dynamics is investigated. Here, we set four levels for the total population size, which are 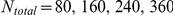, three levels for 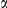, which are 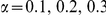, respectively, and the other parameters are taken as 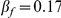, 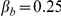, 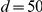, 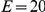 and 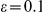. For all simulations in this subsection, the initial proportion of 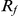-individuals is fixed to be 50%. The simulation results with different numbers of predator attacks are plotted in Figure 2. It is easy to see that: (i) for the situation with small total population size (i.e. 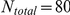), the fearful individuals will be favored (i.e. 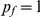) if the number of predators attacks is equal to or bigger than 20 (i.e. 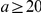) for all three levels of 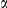 (see Figure 2A), and when 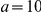, the value of 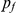 will decrease with the increase of 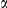; (ii) for the situation with 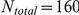, the fearful individuals will be favored if the number of predators attacks is in the interval 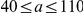 for all three levels of 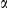, and the value of 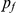 will decrease with the decrease of 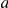 if 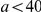 and with the increase of 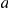 if 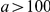, where for both 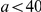 and 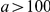 the low level of 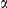 will be helpful to the fearful individuals (see Figure 2B); (iii) for the situation with 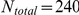, the effect of the number of predators attacks on 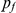 is symmetric about 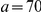 when 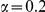 and 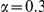, i.e. at 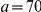 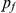 has the maximum 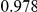 for 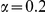 and 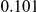 for 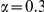, and, similar to the situation with 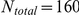, for 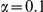, 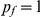 if 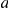 is in the interval 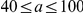 and 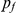 will decrease with the decrease of 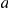 if 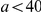 and with the increase of 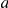 if 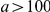 (see Figure 2C); and (iv) for the situation with 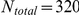, only when 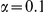 the effect of 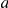 on 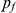 is symmetric about 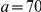 and the maximum of 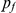 at 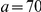 is 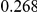, and when 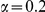 (or 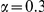), 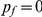 for all possible 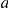 (see Figure 2D).

The simulation results in this subsection imply that the effect of predation pressure (i.e. the number of predator attacks)on the evolution of fearfulness and boldness strongly depends on the total population size, i.e. the fates of fearful and bold individuals are mainly determined by the total population size. On the other hand, we can also notice that for the moderate total population size (for example 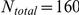 and 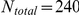), the fearful individuals will be not favored if the number of predators' attacks is small or large, i.e. in our simulations, the effect of 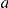 on 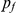 is symmetric about 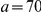 and 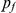 has the maximum at 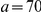.

### Effect of the energy loss per escape

**Figure 3:**
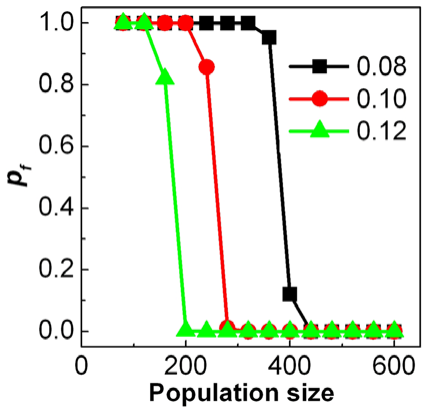
The effect of energy lost per escape on the evolution of fearfulness and boldness where 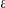 = 0.08, 0.10 and 0.12.

In section 2, we define 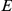 to be the total energy gained during a breeding season and 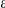 the energy lost per escape. Clearly, the large 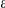 should be always disadvantageous to the fearful individuals since during a breeding season, fearful individuals will take flights more often than bold individuals, and thus fearful individuals will have less energy remained for reproduction. In this subsection, the effect of 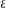 on 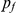 under different total population size is investigated, where we set three levels for 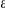, which are 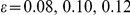, respectively, and the other parameters are taken as 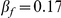, 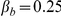, 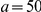, 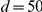 and 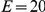. The initial proportion of 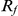-individuals is also fixed to be 50%. The simulation results show clearly that the smaller the value of 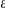 is, the more favored the fearful individuals will be by natural selection, i.e. for 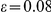, 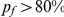 if 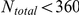; for 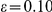, 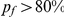 if 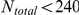; and for 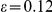, 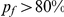 if 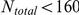 (see Figure 3). This means that although the total population size is the most important factor for the fates of fearfulness and boldness, the small energy loss per escape will make that the fearful individuals have the ability to win the advantage in a large population.

### Effect of the interactions between fearful and bold individuals on the coexistence of fearfulness and boldness

**Figure 4:**
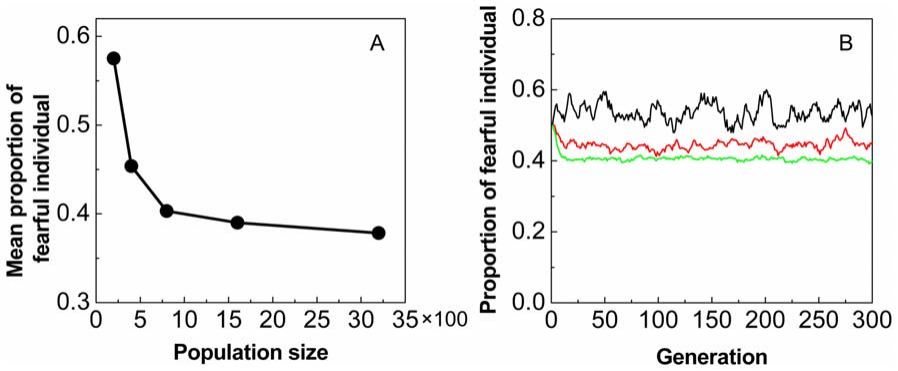
The effect of interactions between individuals on the co-existence of fearfulness and boldness, where the parameters in background fitnesses 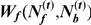 and 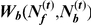 are taken as: 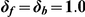, 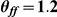, 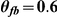, 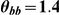, and 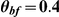. (A) The mean proportions of fearful individuals (for the last 100 generations) decreases with the increase of the total population size. (B) The strength of random fluctuation in the frequency of fearfulness decreases with the increase of the total population size, where black line: 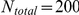; red line: 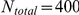; and green line: 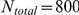.

In this subsection, in order to provide a possible mechanism for the co-existence of fearfulness and boldness, the interactions between fearful and bold individuals (i.e. the background fitnesses of feraful and bold individuals (see Eqs. 7 and 8)) are introduced. The total population size is set five levels, which are 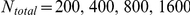 and 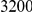, and the parameters 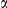, 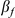, 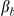, 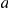, 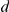, 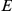 and 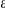 are taken as 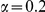, 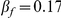, 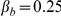, 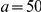, 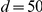, 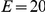 and 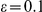, respectively. For the background fitnesses 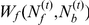 and 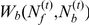 (see Eqs, 7 and 8), we take 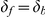, 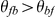 and 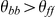 (i.e. for the interactions between individuals, we assume that the effects of 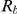-individuals are stronger than that of 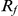-individuals, or generally, individuals would be more aggressive in competition if they risk more when confronted with predators [22]). The stochastic simulation results show clearly that: (a) the co-existence of fearfulness and boldness is possible when the background fitnesses are introduced, i.e. for a given total population size, the frequency of 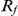 (or 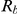) will fluctuates around its mean; (b) the mean proportion of fearful individuals (i.e. the mean frequency of 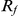) in the co-existence will decrease with the increase of the total population size (see Figure 4A), or the mean proportion of bold individuals will increase with the increasing of the total population size; and (c) the strength of random fluctuation in the frequency of 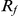 (or 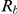) will decrease with the increase of the total population size (see Figure 4B).

## Discussion

As pointed out in the section of introduction, Ji et al. investigated a deterministic model for the evolutionary dynamics of fearfulness and boldness [16]. Their main results show that for large population size bold individuals have a higher expected fitness than fearful individuals, and for small population size fearful individuals have a higher expected fitness. Thus, we firstly focus our attention on how the evolutionary dynamics of both fearfulness and boldness is affected by the total population size (where we ignore temporarily the effect of the interactions between individuals on the dynamics). The simulation results support the theoretical analysis of Ji et al. [16] generally. Our result also implies that for both fearful and bold individuals, the trade-off between survival and reproduction is mainly determined by the total population size, and is independent of the population structure. According to Maynard Smith [23], for only two phenotypes, i.e. 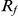 and 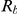, 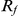 can be considered to be an ESS if the population size is small, and 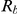 is an ESS if the population size is large.

For the effect of predation pressure, the simulation result reveals that although total population size affects the evolutionary processes significantly, the number of predator attacks also play an important role in a relatively moderate or small population that neither too many nor few attacks are favored by the fearful individuals. Clearly, although the bold individuals will take higher predation risk than the fearful individuals, the bold individuals will also reserve more energy for reproduction. Thus, relatively smaller number of predator attacks will lead to that the bold individuals have a higher expected fitness. Conversely, if the intensity of predator attacks is relatively stronger, the predation risk will be shared among fearful and bold individuals, and the fearful individuals will be unable to maintain a higher expected reproductive success because of its big energy expense in the frequent escapes, i.e. the advantage of fearful individuals in survival cannot compensate its disadvantage in reproduction. However, we have to say that in general the effect of predation pressure also depends strongly on the total population size.

The simulation result shows also that species-specific energy loss per escape is important during the evolution of personality in a population that relatively small value enables that the fearful individuals have the ability to supplant the bold one even in a large population.

Finally, we develop a possible mechanism for the co-existence of fearfulness and boldness. Notice that our basic model considers only two pure strategies in a very simple world with only one available ecological niche. This is why the stable coexistence of fearfulness and boldness is impossible if we ignore the other possible ecological mechanisms in our model. The classical competitive exclusion principle shows that two species competing for the same resources cannot coexist if other ecological factors are constant [24]. While the interactions between fearful and bold individuals (i.e. the background fitnesses of fearful and bold individuals) are introduced, the main result shows that the co-existence of fearfulness and boldness is possible. Moreover, the mean proportion of fearful individuals will decrease with the increase of the total population size. Clearly, this result is also consistent with the theoretical analysis of Ji et al. [16], i.e. the evolution of fearfulness-boldness should be population size-dependent, the fearful behavior will be favored by the natural selection in a small population, and, conversely, the bold behavior will be favored in a large population. We also noticed that some empirical observations had shown that both fearful and bold individuals can be found in a real population, such as [25] and [26]. So, a possible mechanism behind the co-existence of fearfulness and boldness should be that the evolution of fearfulness and boldness not only depends on predator attacks (or nonlethal disturbance) but also depends on their ability in competition for some limited resources. Specifically, as proposed by Thingstad [27], this coexisting phenomenon including the framework of an Lotka-Volterra type model might be caused by imposing a cost on the winner in the modeling, i.e. “killing the winner”, where “winner” refers to the more active population [28]. Our model shows that this mechanism for the co-existence of fearfulness and boldness is possible.

## Supporting Information

Appendix S1 **The ODD Protocol for the individual-based model and the stochastic simulation program in Matlab.**
(PDF)
