# Supplementary material for: Evolutionary Dynamics of Fearfulness and Boldness: A Stochastic Simulation Model
Source: PLoS One. 2012 Mar 8;7(3):e32258. doi: 10.1371/journal.pone.0032258 (PMC3297597; doi:10.1371/journal.pone.0032258)
Supplement: Appendix S1 — The ODD Protocol for the individual-based model and the stochastic simulation program in Matlab. (PDF) [file pone.0032258.s001.pdf]

Appendix S1. The ODD Protocol for the individual-based model and the stochastic simulation program in Matlab

Nan Lu[1,2], Ting Ji[1]*, Jia-Hua Zhang[1], Yue-Hua Sun[1] and Yi Tao[1]

[1]Group of Theoretical Biology, Group of Avian Ecology,

Key Laboratory of Animal Ecology and Conservation Biology,

Institute of Zoology, Chinese Academy of Sciences,

Beijing, P.R. China

[2] Graduate University of Chinese Academy of Sciences,

Beijing, P.R. China

1 ODD protocol

The ODD protocol [1, 2] for our individual-based model is given below.

(a) Purpose. The main goal of our model is to confirm the theoretical results of Ji et al. [3], i.e. whether the fearful and bold individuals could coexist or not, and whether the effect of risk sharing will play an important role on the evolution of fearfulness and boldness. On the other hand, we also interested in the sensitivity of different parameters in the evolutionary dynamics of fearful and bold behaviors. Finally, we try to give a possible mechanism for the co-existence of fearfulness and boldness in nature.

(b) State variables and scales. Here, for simplicity, we consider an asexual population undergoing both predatory attacks and non-lethal disturbing events [3, 4]. Only two possible behavior traits can be exhibited when the population is disturbed, one is fearfulness

*Author for correspondence, and e-mail: jiting@ioz.ac.cn

and the other boldness (detailed descriptions can be found in the main text). For our model, the other variables and scales are given below:

(i) The population is composed of two pure strategists, R_f- and R_b-individuals. The offspring will have the same phenotype with their mother. The initial numbers of them are denoted as N_f and N_b.

(ii) All individuals are assumed to have the same maximum natural lifespan (or maximum survival age), denoted by T year old. The individual's maturity age for reproduction is one year old.

(iii) During a breeding season, the number of real predatory attacks is assumed to be a constant, denoted by a, and, similarly, the number of simple disturbing events is denoted by d.

(iv) The relative probability that a R_f-individual is selected by the predators, compared with a R_b-individual is denote as $\alpha \in (0, 1)$. We also use β_f to denote the probability that a R_f-individual is captured when selected by the predator, and β_b the probability that a R_b-individual is captured when selected.

(v) During a breeding season, each individual would gain a certain amount of energy (E), and each escape would consume the energy of ε.

(c) Process overview and scheduling. According to the variable definitions and assumptions in (b) (see also the main text), the corresponding stochastic simulation (see also Section 2) is conducted:

(i) At the starting of the process, both the R_f- and R_b-individuals are set averagely among different ages, which make up a population.

(ii) During each breeding season, the population would experience the predator attacks and disturbances. The specific probability that a R_f- or R_b-individual is killed by the predators is shown in the main text (see assumption (iii)).

(iii) The reproduction is assumed only occurs at the end of each breeding season. The total number of offspring born in a breeding season is exactly equal to the total number of the dead individuals due to the predator attacks and the limitation of individual's lifespan, according to the assumption that the total population size is assumed to be kept constantly at the end of each breeding season.

(iv) The numbers of R_f- and R_b-individual among the new born individuals are shown in the main text (see assumptions (iv) and (v)).

For given the initial condition (i.e. the initial proportions of R_f- and R_b-individuals), we run the simulation until the population becomes a pure strategy population (i.e. R_f-population, or R_b-population). We repeat this process 1000 times and then count the times that the R_f-population occurs, denoted by C_f, or the frequency that the R_f-population occurs, denoted by $p_f = C_f/1000$.

(d) Design concepts.

(i) Emergence. The model was designed to explore the evolution of animal personality, i.e. fearful or bold. Population dynamics emerge from different personalities of the individual, and from the environment, i.e. attacks and disturbances.

(ii) Fitness. Fitness is measured automatically at the end of each breeding season, which directly determine the number of newly born R_f- and R_b-offspring.

(iii) Sensing. During each attack, the predator is assumed to know, without error, the personality of each individual in the population. Furthermore, the predators are assumed to be able to randomly sample from the population.

(iv) Interaction. The competitive interaction between fearful and bold individual is considered in the submodel (ii).

(v) Stochasticity. Stochasticity is incorporated into two aspects of this model, that are the target individual selection and the killing probability of the selected individual.

(e) Initialization. The population is initialized with both R_f- and R_b-individuals. The predatory attacks and disturbances will occur during each breeding season. The variables listed in (b) are initially taken as $\alpha = 0.2$, $\beta_f = 0.17$, $\beta_b = 0.25$, $a = 50$, $d = 50$, $E = 20$ and $\varepsilon = 0.1$.

(f) Input. Following the initialization, further inputs are not required generally. However, since we also aimed to illustrate the effects of population size, predation risks and energy lost per escape on evolutionary dynamics of fearfulness and boldness, we run the models with different values of N_{total}, a and ε as the other inputs.

(g) Submodels.

(i) In order to explore the effect of risk sharing on the evolution of fearfulness and boldness, we consider the situation where it is assumed that the number of newly born R_f- and R_b-individuals are independent of the numbers of the alive R_f- and R_b-individuals at the end of each breeding season, or that the probability that the fearful individuals (or bold individuals) are selected by the predators is independent of the population structure (i.e. it is frequency-independent). The more detail about this model can be found in the main text.

(ii) In order to explore the potential effect of competitive interactions between R_f- and R_b-individual, we also do simulations embody the frequency-dependent background fitness (see eq. 7 in the main text), and thus revise the corresponding numbers of the newly born R_f- and R_b-offspring according to the eq. 8 (see in the main text).

2 Stochastic simulation program in Matlab

In this section, our stochastic simulation program in Matlab is given below.

```
function [pf]=fearfulbold(f,b,e,s,a,d,aa)
% pf represents the frequency of fearful population occurs;
% f and b represent the initial numbers of fearful and bold individual;
```

```
% e represents the whole energy gained within a breeding season;
% s represents the energy lose per flight;
% a and d represent the numbers of real attack and disturbance within
% a breeding season
% aa represents the selection probability alfa
R=zeros(1000,1);
for t=1:1000
% The iteration number of the whole programme
    fp=f;bp=b;
    fb=fp+bp;h=fp/4;k=bp/4;
    A=zeros(3000,1);B=zeros(3000,1);
    for i=1:h
    % define the initial age of each individual
        A(i,1)=4;
        A((h+i),1)=3;
        A((2*h+i),1)=2;
        A((3*h+i),1)=1;
    end
    for j=1:k
        B(j,1)=4;
        B((j+k),1)=3;
        B((j+2*k),1)=2;
        B((j+3*k),1)=1;
    end
    for m=1:3000
    % the number of breeding season
        n1=0;n2=0;q1=0;q2=0;
```

```
for i=1:a
% the attacks begin
    y=fp*aa/(fp*aa+bp);
    c=rand(1,1);
    if c<y
    % check that whether the selected individual is fearful
        v=rand(1,1);vv=round(5*v);
        if vv(1,1)==2
        % check that whether the target individual was killed
        % the dead individuals will be removed from the population
            u=rand(1,1);uu=round((fp-1)*u);
            for ii=(uu(1,1)+1):(fp-1)
                A(ii,1)=A((ii+1),1);
            end
            A(fp,1)=0;
            fp=fp-1;
            if fp<1
            % check that whether all of the fearful
            % individuals are dead
                fp=0;
                bp=fb;
                break;
            end
            n1=n1+1;
        end
    else
    % the predator chooses a bold individual
```

```
            w=rand(1,1);ww=round(3*w);
            if ww(1,1)==2
                x=rand(1,1);xx=round((bp-1)*x);
                for j=(xx(1,1)+1):(bp-1)
                    B(j,1)=B((j+1),1);
                end
                B(bp,1)=0;
                bp=bp-1;
                if bp<1
                    fp=fb;
                    bp=0;
                    break;
                end
                n2=n2+1;
            end
        end
    end
    l=1;p=1;
    for i=1:fp
    % check the age of the individuals.
    % The individuals achieved their age limitation will be
    % sent to death.
        if A(i,1)>3
            A(i,1)=0;
            q1=q1+1;
        end
    end
```

```
for i=1:fp
% remove the fearful individuals achieved age limitation
    if A(i,1)>0
        A(l,1)=A(i,1);
        l=l+1;
    end
end
if fp>l
    for i=l:fp
        A(i,1)=0;
    end
end
fp=l-1;
if fp<1
    fp=0;
    bp=fb;
    break;
end
for j=1:bp
    if B(j,1)>3
        B(j,1)=0;
        q2=q2+1;
    end
end
for j=1:bp
% remove the bold individuals achieved age limitation
    if B(j,1)>0
```

```
            B(p,1)=B(j,1);
            p=p+1;
        end
    end
    if bp>p
        for j=p:bp
            B(j,1)=0;
        end
    end
    bp=p-1;
    if bp<1
        bp=0;
        fp=fb;
        break;
    end
    nq=n1+n2+q1+q2;
    mm=1-exp((-0.1)*(e-(a+d)*s));
    % the fitness gained of the fearful individual at the end
    % of the breeding season
    nn=1-exp((-0.1)*(e-a*s));
    % the fitness gained of the bold individual at the end
    % of the breeding season
    f1=round(nq*(mm*fp)/((mm*fp)+(nn*bp)));
    % the number of the newly born fearful individuals
    b1=nq-f1;
    for i=1:(fp+f1)
    % the individual age will be set as increased by one year old.
```

```
            A(i,1)=A(i,1)+1;
        end
        for j=1:(bp+b1)
            B(j,1)=B(j,1)+1;
        end
        fp=fp+f1;
        bp=bp+b1;
        if fp==0
            bp=fb;
            break;
        elseif bp==0
            fp=fb;
            break;
        end
    end
    R(t,1)=fp/(fp+bp);
    disp(fp/(fp+bp));
    % the proportion of the fearful individual
end
pf=0;
for t=1:1000
    pf=pf+R(t,1);
end
pf=pf/1000;
```

References

[1] Grimm V, Berger U, Bastiansen F, Eliassen S, Ginot V, et al. (2006)A standard protocol for describing individual-based and agent-based models. Ecol Model 198: 115-126.

[2] Grimm V, Berger U, DeAngelis DL, Polhill JG, Giske J, et al. (2010) The ODD protocol: A review and first update. Ecol Model 221: 2760-2768.

[3] Ji T, Zhang B, Sun Y, Tao Y (2009) Evolutionary dynamics of fearfulness and boldness. J Theo Biol 256: 637-643.

[4] Sirot E (2007) Game theory and the evolution of fearfulness in wild birds. J Evol Biol 20: 1809-1814.
